# Diffusion-weighted MR imaging and assessment of ovarian carcinomas with vaginal deposit accidentally detected during pregnancy

**DOI:** 10.1259/bjrcr.20150411

**Published:** 2016-10-14

**Authors:** Soha Hamed, Rasha Kamal, Mamdouh Sheeba, Marwan Mohamed El-Toukhy, Sahar Mansour

**Affiliations:** ^1^Faculty of Medicine, Radiology Department (Women’s Imaging Unit), Cairo University, Cairo, Egypt; ^2^Obstetrics and Gynecology Department, Cairo University Hospitals, Cairo, Egypt

## Abstract

Ovarian cancer during pregnancy is a complex situation that endangers the lives of both the pregnant female and the fetus. We present a 40-year-old pregnant female in the third trimester with bilateral undifferentiated ovarian adenocarcinoma and vaginal metastasis. The case was evaluated by ultrasound and MRI supported with diffusion-weighted sequence.

## Introduction

Gynaecological cancer during pregnancy is a demanding challenge because cancer as well as its treatment may affect not only the pregnant female in general but may also directly affect the reproductive tract and the fetus as well.^1^ Pregnancy affects the management protocol of cancer, whereas cancer affects the management and outcome of pregnancy. The incidental finding of an adnexal mass during pregnancy is becoming more feasible with the increased use and quality of ultrasound imaging. The overall incidence of adnexal masses in pregnancy can vary greatly based on the study population, use of ultrasound and gestational age at presentation.^2,3^ It is reasonable to estimate that clinicians can expect to encounter adnexal masses in 2–10% of pregnancies.^3^ A functional cyst is the most common adnexal mass found during pregnancy, similar to the non-pregnant state. A corpus luteum persisting into the second trimester accounts for 13–17% of all cystic adnexal masses.^4^ However, the differential diagnosis throughout pregnancy also includes the following: benign masses such as a dermoid cyst (7–37% incidence), serous cystadenoma (5–28% incidence), mucinous cystadenoma, endometrioma, hydrosalpinx, heterotopic pregnancy and leiomyoma, with an incidence of 1–2.5%.^5^ Ovarian cancer occurs in approximately 1 in 12,500–25,000 pregnancies.^6^Ovarian cancer spreads primarily by intraperitoneal implantation of exfoliated cancer cells, lymphatic dissemination or haematogenous spread. Very rarely, it metastasizes to the cervix, vulva and vagina; this type of metastases present a diagnostic challenge to radiologists, gynaecologists and pathologists.^7^

## Case report

**Figure 1.**
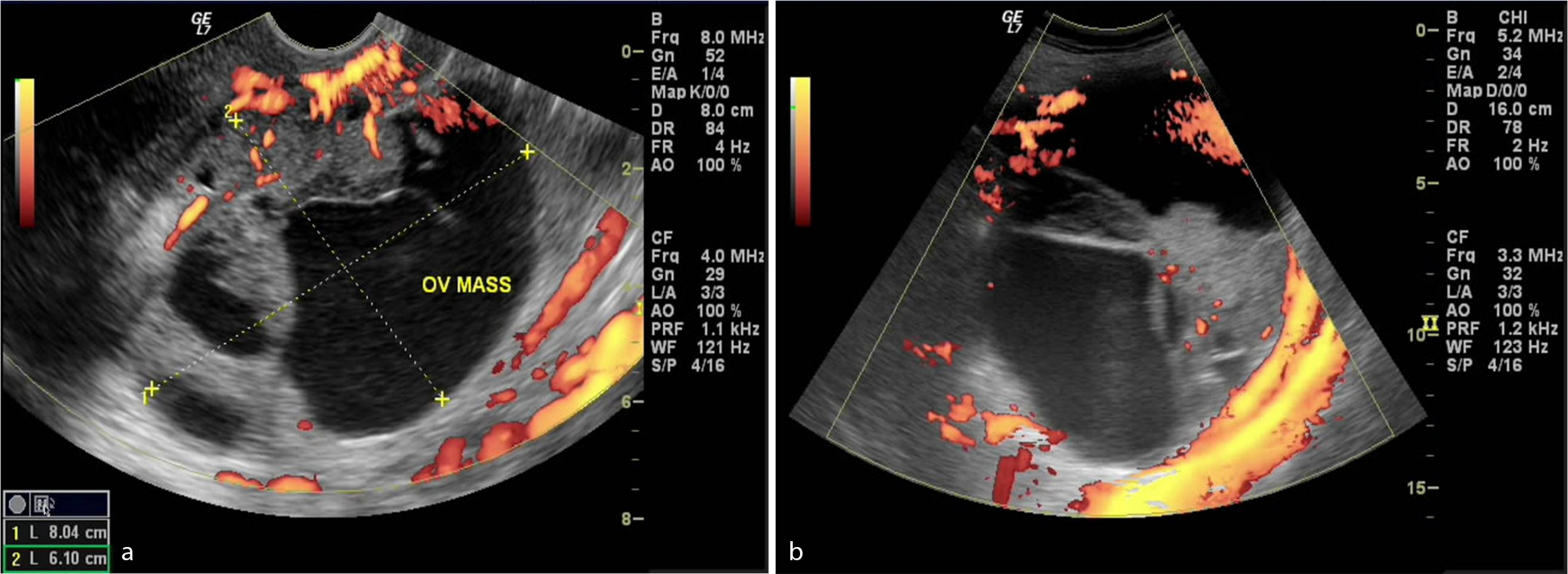
(a and b) Ultrasound with power Doppler imaging shows bilateral complex ovarian masses. OV, ovarian.

**Figure 2.**
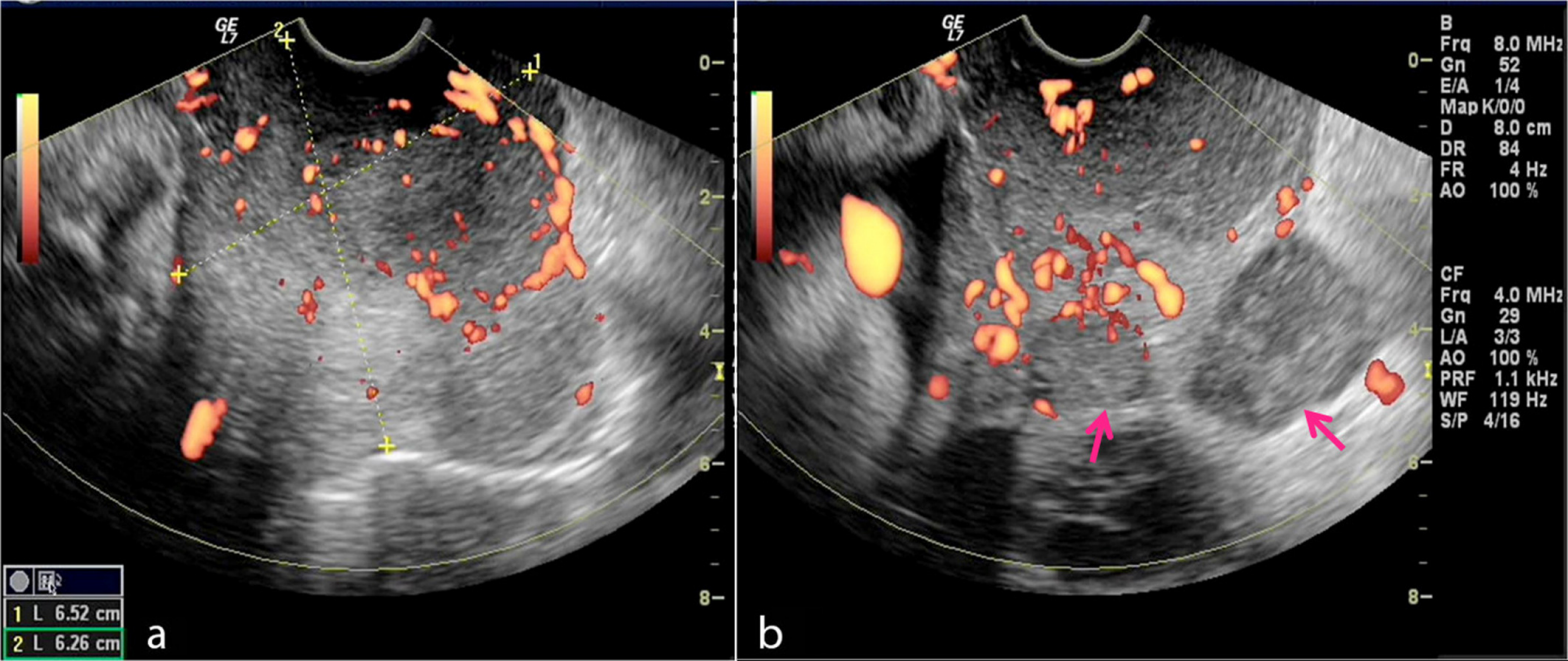
Transvaginal ultrasound with power Doppler imaging shows a bulky highly vascular cervix (a) and polypoidal masses seen in the upper vagina. (b) Peritoneal deposits are seen in the Douglas pouch (arrow).

**Figure 3.**
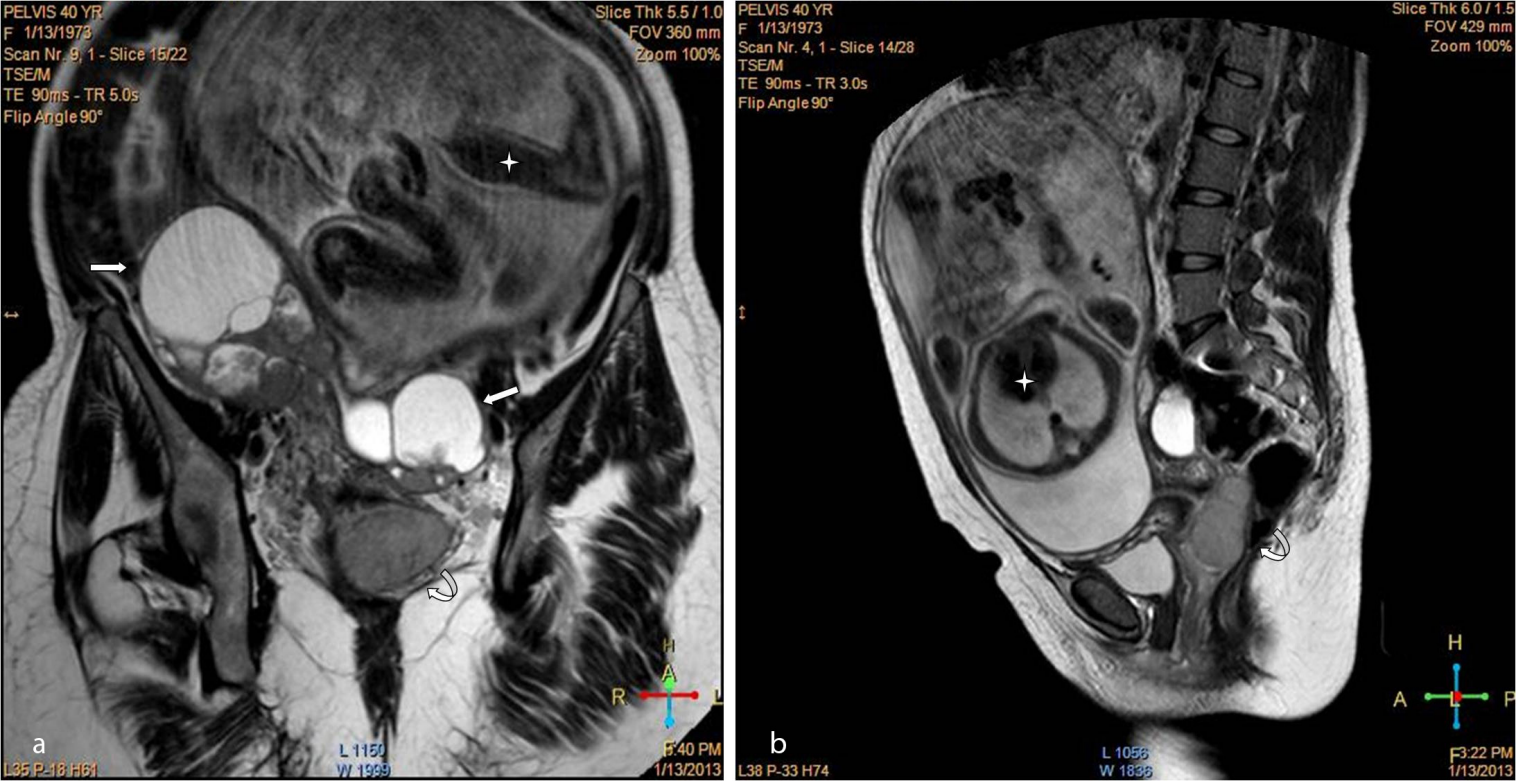
*T*_2_ weighted coronal (a) and sagittal (b) images show bilateral complex cystic mass lesions (straight arrows) and upper vaginal mass (curved arrow). A noteworthy feature is the fetal parts (asterisks).

**Figure 4.**
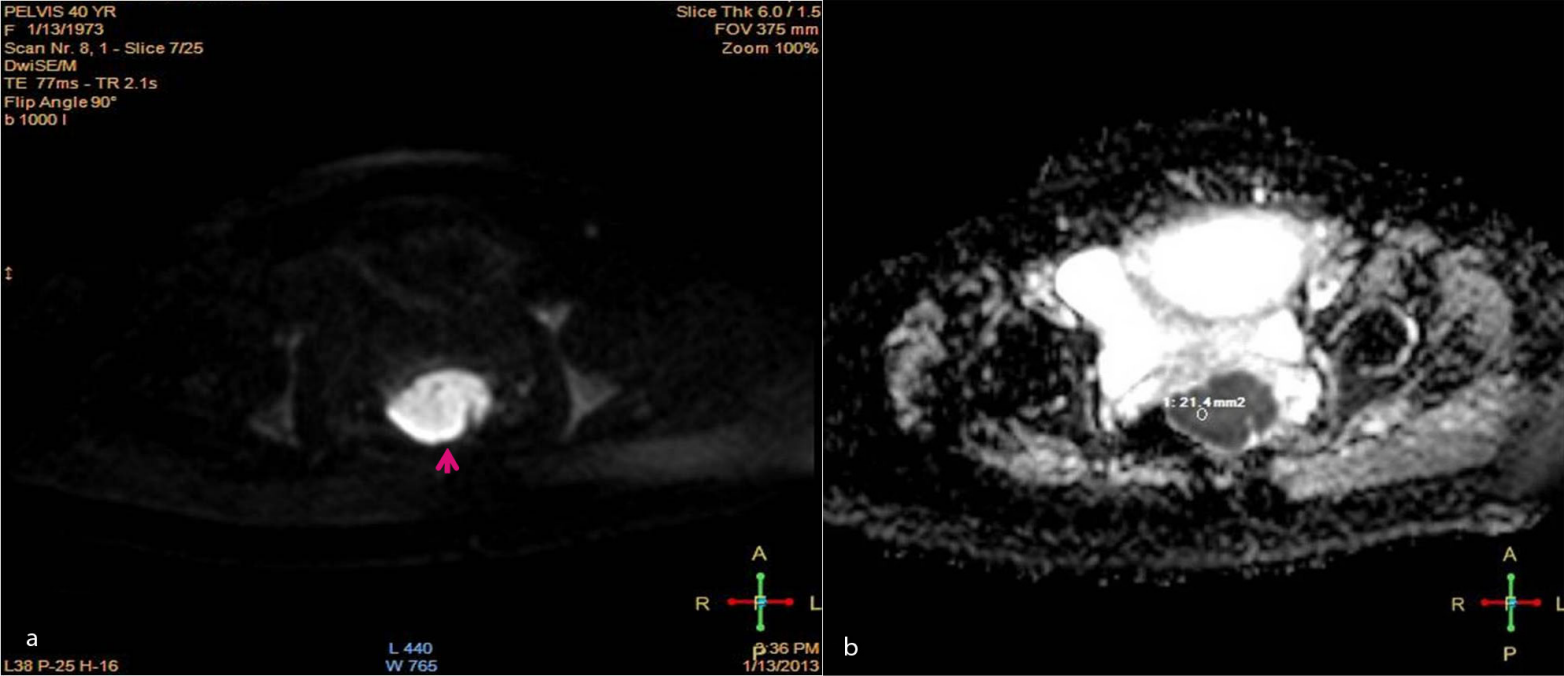
(a) Diffusion-weighted images show restricted diffusion in the upper vaginal mass (arrow). (b) Apparent diffusion coefficient map shows intermediate signal intensity and low ADC value by the applied ROI. These findings add to the sugesstion of malignancy in the vaginal mass.

We report a case of a 40-year-old female (gravida 5 para 4, gestational age 32 weeks) who attended the obstetrics and gynaecology outpatient clinic in our institute. She complained of repeated episodes of vaginal bleeding. Abdominal examination revealed a disproportionately high uterine fundal level that was not matching her corresponding gestational age. Abdominal ultrasound examination showed a viable, single intrauterine 32 weeks fetus. On screening the adnexal regions, bilateral highly vascular complex adnexal masses were identified, measuring 8 × 6 cm² on the right side and 12 × 8 cm² on the left side (Figure 1). Transvaginal ultrasound imaging showed an additional highly vascular polypoidal mass projecting in the upper vagina, indistinct from a rather bulky cervix. Solid peritoneal deposits were also identified in the Douglas pouch (Figure 2). Further evaluation of the pelvis by MRI was requested and a non-contrast study was performed at the same institute after a 1-week interval, which was interpreted by a different set of readers. *T*_2_ weighted images showed findings matching those of the preliminary pelvic ultrasound imaging (Figure 3). Functional data concerning the cellularity and integrity of the cell membranes of the adnexal and vaginal masses was provided by the diffusion-weighted MRI (DWI) sequence. On the DWI sequence, the masses showed restricted diffusion in the form of persistent bright signal intensity and low apparent diffusion coefficient (ADC) values, which strongly favoured likely malignant pathology (Figure 4). Laboratory data were within normal limits apart from a raised CA-125 level. Corticosteroids were administered to ensure fetal lung maturity. The pregnancy was terminated by an elective caesarean section at 34 weeks gestation *via* a midline subumbilical incision. A 2.5 kg living normal fetus was extracted. The abdomen was explored and the bilateral complex adnexal masses were identified, together with the multiple omental metastatic nodules and bloody ascites. The right ovarian mass was attached to the posterior abdominal wall and was inseparable from the sacral promontory. Debulking of the ovarian lesions, panhysterectomy, bilateral salpingo-oophorectomy and omentectomy were performed. Minimal tumour residue that was adherent to bone could not be removed. Vaginal exploration was also performed to remove the polypoidal vaginal component, which was grasped and totally excised. It was completely separable from the cervix. Pathological examination revealed an undifferentiated ovarian adenocarcinoma (grade 3), metastasizing to the upper vagina with clear cervical margins. The patient is now undergoing chemotherapy.

## Discussion

Adnexal masses may be detected during prenatal ultrasound, and ovarian cancer may be suspected during pregnancy. It is a common assumption by both patients and physicians that if ovarian cancer is diagnosed during pregnancy, treatment necessitates sacrificing the well-being of the fetus. However, in most cases, it is possible to offer appropriate treatment to the mother without placing the fetus at serious risk.^8^ Both ultrasound and MRI are relatively safe and widely used during pregnancy.

In the presented case, transvaginal ultrasound was the primary tool that suggested the malignant over benign nature of the adnexal masses and also confirmed their ovarian origin. The presence of solid areas, as well as increased vascularity as determined by Doppler ultrasound, favoured the malignant nature of the lesions. On the other hand, the nature and origin of the vaginal mass could not be clarified by the transvaginal ultrasound examination; there was only a suspicion of unhealthy cervix. An MRI may be performed if the ultrasound does not provide enough information.^9^ It plays an important role in the diagnosis of gynaecological adnexal lesions.^10^ Some morphological and signal intensity features of the lesions on MRI are very important for the differential diagnosis,^11^ but this information may sometimes be non-specific. Many studies have looked at the utility of DWI in the differential diagnosis of benign and malignant gynaecological lesions. In particular, the contributions of DWI and ADC values in differentiating between benign and malignant cystic ovarian and uterine lesions have been evaluated.^12^ In the presented case, contrast injection is contraindicated owing to the pregnancy status of the patient, yet with the aid of DWI, valuable information about the adnexal masses was obtained. The presence of a persistent bright signal of restricted diffusion and low ADC values elicited by the solid component of the adnexal masses confirmed their malignant nature; moreover, proper evaluation of the whole case and proper staging was also possible owing to the detection of the vaginal mass previously missed by ultrasound. MRI examination also showed free cervical margins, which was proven by histopathological examination in contrast to the ultrasound data. Similar lesions have been reported in several previous studies.^7,13,14^ Although primary vaginal cancer is rare, metastatic disease to the vagina is not uncommon, and they often arise from the endometrium, cervix, vulva, ovary, breast, rectum and kidney. Vaginal metastases may occur by direct extension (*eg*, cervix, vulva and endometrium), or by lymphatic or haematogenous spread (*eg*, breast, ovary and kidney).^11^

To conclude, the radiologist and clinicians should be aware of malignant masses encountered in pregnant females. Imaging, including ultrasound and DWI, should be used for proper staging and, consequently, proper management.

## Learning points

1. Ovarian cancer during pregnancy is very rare.
2. Ovarian cancer spreads primarily by intraperitoneal implantation very rarely to the vagina.
3. A large gravid uterus may limit the precise assessment of the genital organs on ultrasound examination.
4. MRI has a wide field of view and multiplanar capability, yet may require contrast injection to confirm/exclude malignancy.
5. DWI provides comparable information to a contrast-enhanced study in the case of ovarian masses during pregnancy.

## Consent

The patient had given an informed consent.
